# Quantitative determination of blended proportions in tobacco formulations using near-infrared spectroscopy and transfer learning

**DOI:** 10.3389/fpls.2025.1617958

**Published:** 2025-08-07

**Authors:** Qinlin Xiao, Ruifang Gu, Li Li, Jing Wen, Xixiang Zhang, Yi Shen, Yang Liu, Lan Xiao, Qinqin Tang, Jun Yang, Yong He, Juan Yang

**Affiliations:** ^1^ Technology Center, China Tobacco Sichuan Industrial Co., Ltd., Chengdu, China; ^2^ College of Biosystems Engineering and Food Science, Zhejiang University, Hangzhou, China

**Keywords:** near-infrared spectroscopy, transfer learning, blended proportions, tobacco silk, quantitative detection

## Abstract

Accurate detection of blending proportions in tobacco formulations is crucial for ensuring the quality consistency and flavor stability of cigarette products. In recent years, modeling approaches based on near-infrared spectroscopy (NIRS) have attracted significant attention for the quantitative analysis of tobacco blending. However, due to variations in tobacco composition and spectral characteristics across different cigarette brands, the generalization ability of NIRS-based models often declines when applied to cross-brand prediction tasks. To address this issue, this study takes the detection of blending proportions of tobacco silk in tobacco formulations as the research focus, and investigates transfer learning strategies aimed at enhancing the cross-brand adaptability of NIRS-based models. A partial least squares regression (PLSR) model was first developed using NIRS data from four different tobacco brands, achieving high prediction accuracy on the combined dataset (RMSEP = 1.20%). However, when the model trained on a single brand was applied to predict other brands, the prediction performance decreased notably. To improve model adaptability, three approaches were explored: Transfer Component Analysis (TCA), Correlation Alignment (Coral), and model updating. The results show that TCA-PLSR achieved substantial reductions in prediction error in most transfer tasks involving large discrepancies in feature distributions. Coral-PLSR demonstrated superior performance in transfer tasks involving similar spectral feature distributions. Additionally, in transfer tasks characterized by substantial distribution differences, the Updated-TCA-PLSR model, which incorporates a small proportion of target domain samples into the source domain before domain adaptation, yielded accurate predictions of tobacco silk blending proportions. These findings demonstrate that transfer learning and model updating offer practical, flexible, and robust approaches for enhancing the performance of NIRS-based models, supporting more accurate and consistent quality control in industrial-scale formulated tobacco production.

## 1 Introduction

The cigarette industry, as a significant component of the global economy, plays a crucial role in contributing to fiscal revenue and employment. Its sustainable development is closely linked to the stability and growth of the broader socio-economic landscape (Yach and Bettcher, 2000; Zhu et al., 2022). According to data from the World Health Organization, China is not only the largest producer but also the largest consumer of tobacco products globally (Zhu et al., 2024). With the continuous rise in consumer expectations for cigarette quality and the industry’s growing emphasis on product consistency and stability, quality control has become a central concern in cigarette manufacturing. In cigarette production, the blending proportions of components in the formulated cut tobacco—such as tobacco silk, cut stem, and expanded tobacco silk, and reconstituted tobacco shred—have a direct impact on the overall quality of the final product. These components determine not only the chemical composition and combustion characteristics of cigarettes but also significantly influence the sensory experience (Chen et al., 2015; Wu et al., 2022). Moreover, an optimized blending ratio enhances resource utilization efficiency and reduces manufacturing costs, thereby improving the overall economic performance of the production process. Therefore, the accurate detection of the blending proportions in formulated cut tobacco is of great significance for ensuring the consistency and stability of cigarette quality, meeting diverse market demands, and promoting the sustainable use of tobacco resources.

Traditional approaches to determining the blending proportions of components in formulated cut tobacco primarily rely on manual weighing and chemical analysis. Manual separation and weighing of each component is highly subjective and inefficient, with results susceptible to operator variability (Bi et al., 2019). While physicochemical analyses—such as total nitrogen, sugar content, and cellulose—have also been used to infer blending ratios, these techniques are time-consuming, labor-intensive, and not suitable for high-throughput industrial applications (Li et al., 2013; Wu et al., 2022). Therefore, there is a critical need for rapid, accurate, and non-destructive methods for component quantification in formulated tobacco.

Near-infrared spectroscopy (NIRS), which utilizes overtone and combination absorptions of hydrogen-containing functional groups (C–H, N–H, O–H), offers a promising solution due to its speed, non-destructive nature, and ability to capture compositional information (Cen and He, 2007; Pu et al., 2020). In recent years, NIRS has received extensive attention in the tobacco industry, particularly in the detection of physicochemical indicators of tobacco leaves (Liang et al., 2022) and tobacco leaf quality grading (Bin et al., 2016; Li et al., 2020; Liu et al., 2020). In the detection of blending proportions in formulated cut tobacco, Liu et al. (2021) applied NIRS combined with linear non-negative regression to determine the proportion of cut stem. Using orthogonal partial least squares discriminant analysis, pure cut stem, pure tobacco silk, and formulated cut tobacco samples containing different blending proportions of cut stem, achieving relative errors of less than 5% between predicted and actual values, are successfully classified. Similarly, Wu et al. (2022) utilized NIRS to analyze the proportion of cut stems. By employing a partial least squares regression model, rapid quantification of the cut stems blending ratio was achieved, with relative errors within 3.88% for external validation samples. These studies demonstrate that NIRS enables rapid and accurate analysis of blending proportions in formulated cut tobacco. However, existing NIRS models are typically developed and validated within a single brand or formulation context, limiting their generalizability across different tobacco products. Variations in raw material origin, processing techniques, and formulation ratios across brands can significantly affect spectral characteristics, thereby reducing model robustness. The conventional approach to addressing this challenge involves recollecting samples and retraining new models whenever conditions change (Xiao et al., 2022), but this process is time-consuming and labor-intensive, making it difficult to meet industrial demands for rapid detection and quality monitoring. Therefore, enhancing model transferability to enable adaptation to spectral data from different cigarette brands and batches has become a critical issue that needs to be urgently addressed.

Transfer learning has emerged as a significant branch of machine learning, allowing models to leverage knowledge learned from one domain or task to improve performance in a different but related domain or task (Zhuang et al., 2020; Tan et al., 2022). This approach provides an effective means to enhance the generalization ability and adaptability of models, particularly in situations with limited labeled data. Among various transfer learning strategies, feature-based domain adaptation methods such as transfer component analysis (TCA) and correlation alignment (Coral) have shown promising results. These methods aim to reduce distributional discrepancies between source and target domains by aligning data representations in a common feature space, thereby improving model stability and cross-domain applicability. Furthermore, model updating strategies—by incrementally introducing a small number of new samples into the training set—can enable gradual adaptation to changing data distributions, leading to improved prediction accuracy and robustness. Such approaches have been successfully applied in spectral analysis tasks across various domains, including agriculture and food quality assessment, where spectral data often exhibit high dimensionality and domain shift issues (Pan et al., 2011; Wan et al., 2020; Tao et al., 2022; Wan et al., 2022; Pan et al., 2024a; Wang et al., 2025).

Despite the progress in transfer learning and spectral analysis, to date, no research has been reported on applying transfer learning strategies to improve model adaptability and generalization in the field of blend ratio detection for formulated cut tobacco. Current studies on NIRS-based detection models for tobacco primarily focus on specific datasets, limiting their applicability to broader industrial scenarios involving multiple cigarette brands. Therefore, this study aims to explore the potential of integrating transfer learning strategies into blend ratio detection models to enhance their cross-brand applicability. Taking the detection of tobacco silk proportion in formulated cut tobacco across different cigarette brands as a case study, this research aims to: (1) Analyze the spectral characteristics of formulated cut tobacco from various brands to understand inter-brand differences; (2) Evaluate the feasibility of building a robust NIRS-based model for detecting tobacco silk proportions; (3) Investigate the limitations of directly applying models trained on a single brand to other brands; and (4) Investigate the feasibility of integrating TCA, Coral, and model updating strategies to enhance the transferability of blend ratio detection models across different cigarette brands. This work seeks to provide a practical and scalable solution for quality control and monitoring in the cigarette industry by advancing the methodological framework for spectral analysis of formulated cut tobacco.

## 2 Materials and methods

### 2.1 Sample preparation

In this study, raw materials of formulated cut tobacco were collected from four cigarette brands (#1, #2, #3, and #4) provided by China Tobacco Sichuan Industrial Co., Ltd. The materials included tobacco silk, cut stem, expanded tobacco silk, and fermented cut stem. Specifically, Brand #1 consisted of tobacco silk, cut stem, fermented cut stem, and expanded tobacco silk; Brand #2 included tobacco silk and fermented cut stem; Brand #3 contained tobacco silk and cut stem; and Brand #4 comprised tobacco silk and cut stem. Notably, while the tobacco silk components differed across brands, the cut stem, fermented cut stem, and expanded tobacco silk were the same for all brands. All raw materials were dried at 45°C for 4 hours and then ground into fine powders (60 mesh). The powders were subsequently conditioned at 22°C and 60% relative humidity until their moisture content reached 6–8%, after which the prepared materials were sealed and stored.

To simulate the variations encountered in actual cigarette production, the blending proportions of the samples were adjusted within defined ranges based on the standard formulations of each brand, as outlined in **Table 1**. According to the designed ratio, precise quantities of each powdered raw material were weighed to obtain a total mass of around 20 g. During the weighing process, the mass of each component and the total sample mass were accurately recorded. Based on this information, the actual blending ratio of each sample was calculated. The weighed powders were then thoroughly homogenized using a standardized mixing protocol to ensure uniform distribution of each component, thereby enhancing the representativeness and consistency of the blended samples.

**Table 1:** Number of samples and blending ratio ranges for each brand.

Brand	Number of samples	Tobacco silk(%)	Cut stem (%)	Fermented cut stem (%)	Expanded tobacco silk (%)
#1	210	59-100	0-20	0-20	0-25
#2	199	75-100	—	0-25	—
#3	200	75-100	0-25	—	—
#4	183	75-100	0-25	—	—

“—” indicates that the component is not present in the formulation for the corresponding brand.

### 2.2 Spectra acquisition

The near-infrared spectra of the samples were collected using an Antaris™ II Fourier transform near-infrared spectrometer (Thermo Fisher Scientific, USA). A suitable amount of powdered tobacco sample was taken using a sampling spoon and placed into a clean sampling cup. The sample was evenly leveled and gently compressed using a sample press to ensure a minimum thickness of 10 mm. The sampling cup was then positioned on the rotating stage of the NIR spectrometer for spectral acquisition. Each sample was scanned twice, and the average spectrum was used for subsequent analysis. The spectrometer parameters were set as follows: a scanning range of 10,000 cm^-1^ to 4,000 cm^-1^, a spectral resolution of 8 cm^-1^, and 64 scans per measurement.

### 2.3 Data analysis methods

#### 2.3.1 Principal component analysis

Principal Component Analysis (PCA) is a widely used statistical method for dimensionality reduction and feature extraction in high-dimensional data. The underlying principle of PCA is to perform a linear transformation that projects the original high-dimensional variables onto a new set of orthogonal components, known as Principal Components (PCs), in such a way as to maximize the variance in the data while minimizing redundant information (Greenacre et al., 2022). The variance explained by each principal component quantifies the proportion of the total variability in the data attributed to that component, reflecting its ability to explain the underlying features of the dataset. PCA effectively identifies the key structural features of the data, reduces noise interference, and enhances the interpretability of the analysis. In this study, PCA is employed to reduce the dimensionality of the spectra, facilitating the visualization and analysis of the spectral characteristics of formulated cut tobacco from different cigarette brands.

#### 2.3.2 Regression model

The regression model is widely used in the fields of statistics and machine learning to establish mathematical relationships between a dependent variable and one or more independent variables. Through regression analysis, the correlation between the independent variable *x* and the response variable *y* can be quantified, allowing for the construction of a regression model *f(x)* that enables the prediction of corresponding *y* values from new samples based on their *x* values (Xiao et al., 2020). In the field of spectral analysis, regression models are employed to analyze the relationship between spectral data *x* and target properties *y*, enabling the prediction of physicochemical parameters of unknown samples based on their spectral input.

In this study, partial least squares regression (PLSR) was employed to construct a predictive model relating the spectral data of formulated cut tobacco samples to the blending proportions of tobacco silk. PLSR is a multivariate regression technique based on latent variable decomposition, which is particularly effective in handling high-dimensional spectral data, multicollinearity among variables, and limited sample sizes. By simultaneously extracting relevant information from both the independent and dependent variables, PLSR aims to maximize the covariance between the two data matrices while reducing dimensionality. This property enables the model to capture the most informative components for prediction, thereby improving its generalization ability and robustness (Huang et al., 2017; Shao et al., 2024). To prevent overfitting and enhance model stability, 10-fold cross-validation was adopted to determine the optimal number of latent variables.

#### 2.3.3 Transfer component analysis

Transfer Component Analysis (TCA) is an unsupervised feature transformation-based transfer learning method that aims to reduce distributional divergence between source and target domains by learning a common latent subspace (Pan et al., 2011). Specifically, TCA maps the source and target datasets with different distributions into a reproducing kernel Hilbert space (RKHS), where it minimizes the distance between their data distributions while preserving intrinsic data structures (Panigrahi et al., 2021). The distance between distributions is quantified using maximum mean discrepancy (MMD), a non-parametric metric defined in RKHS. In this study, TCA is applied to the transfer task of blending ratio prediction for formulated cut tobacco across different cigarette brands. Specifically, spectral data from the source domain (i.e., samples from one brand) and target domain (i.e., samples from a different brand) are jointly mapped into the learned subspace. A regression model is then trained on the transformed source domain features and subsequently used to predict the blending proportions of the target domain samples. In this work, the primal kernel type was selected, and the target dimension was adjusted to 30.

#### 2.3.4 Correlation alignment

Correlation alignment (Coral) is an unsupervised transfer learning method based on statistical moment matching. The core idea is to minimize the covariance matrices of the source and target domain features, thereby reducing the domain shift and improving the applicability of the model to the target domain (Sun et al., 2016, 2017). Specifically, Coral first calculates the covariance matrices of the source and target domain data, then minimizes the Frobenius norm difference between the two domains, solving for a linear transformation matrix. This matrix transforms the source domain features to make the covariance matrix of the source domain data as similar as possible to that of the target domain data. In this study, Coral is applied to the transfer task of detecting the blending ratio of formulated cut tobacco for different cigarette brands. Using the Coral method, the spectral data from the source domain brand are transformed, and a regression model is constructed based on the transformed source domain features to predict the target domain brand.

#### 2.3.5 Updated model development

Model updating is a model transfer strategy that incorporates a small portion of target domain data into the source domain to enhance model adaptability. Prior studies have demonstrated that introducing even a limited amount of target domain data into the source domain before model training can improve the model’s predictive performance on the target domain (Tao et al., 2022; Wan et al., 2022; Pan et al., 2024b). This strategy enables the model to simultaneously learn the underlying patterns of the source domain while adapting more effectively to the distributional characteristics of the target domain. In this study, we investigate the feasibility of model updating as a strategy to enhance prediction performance and further explore its integration with domain adaptation methods (TCA and Coral). Two scenarios are considered. In the first scenario, only model updating is applied: a small portion of target domain samples is incorporated into the source domain to form an updated source domain, while these samples are simultaneously excluded from the original target domain to construct a new, disjoint target domain. PLSR models are then built using the updated source domain and used to predict the new target domain. In the second scenario, model updating is combined with TCA or Coral: the updated source and modified target domains (as described above) are first used to perform TCA or Coral-based domain alignment. Subsequently, PLSR models are constructed on the transformed source domain and used to predict the new target domain.

### 2.4 Model evaluation

To assess the performance of the tobacco silk blending ratio detection models constructed based on all cigarette brands, this study first merged the samples from all brands and sorted them according to the blending proportions of tobacco silk. For all the sorted samples, every three consecutive samples were grouped, with the first and third samples forming the training set and the second sample serving as the prediction set, resulting in a 2:1 ratio of calibration to prediction samples. To evaluate the transfer performance of the model between different brands, this study used samples from a single brand as the source domain dataset (calibration set), and samples from another brand as the target domain dataset (prediction set), designing a total of 12 transfer tasks. Considering the data distribution differences between the source and target domains, several transfer strategies were introduced to enhance the model’s generalization ability and transferability: TCA, Coral, data updating, and data updating combined with TCA or Coral.

Model performance was evaluated based on the root mean squared error of prediction (RMSEP). In the application of transfer strategies, the transformed target domain was used as the prediction set. Additionally, to comprehensively measure the model’s robustness, the mean absolute error (MAE) and Pearson correlation coefficient for the prediction set (*R_p_*), as well as the root mean squared error of calibration (RMSEC) and Pearson correlation coefficient for the calibration set (*R_c_*), were also used as evaluation metrics to provide a more comprehensive assessment of model performance. The formulas for these metrics can be found in the literature (Naser and Alavi, 2020).

### 2.5 Software tools

The model development and assessment, as well as PCA, were performed in MATLAB R2019b (The Mathworks, Inc., Natick, MA, USA). All of the graphs were drawn using MATLAB R2019b (The Mathworks, Inc., Natick, MA, USA).

## 3 Results and discussion

### 3.1 Distribution of blending proportions of tobacco silk across different cigarette brands

**Figure 1:**
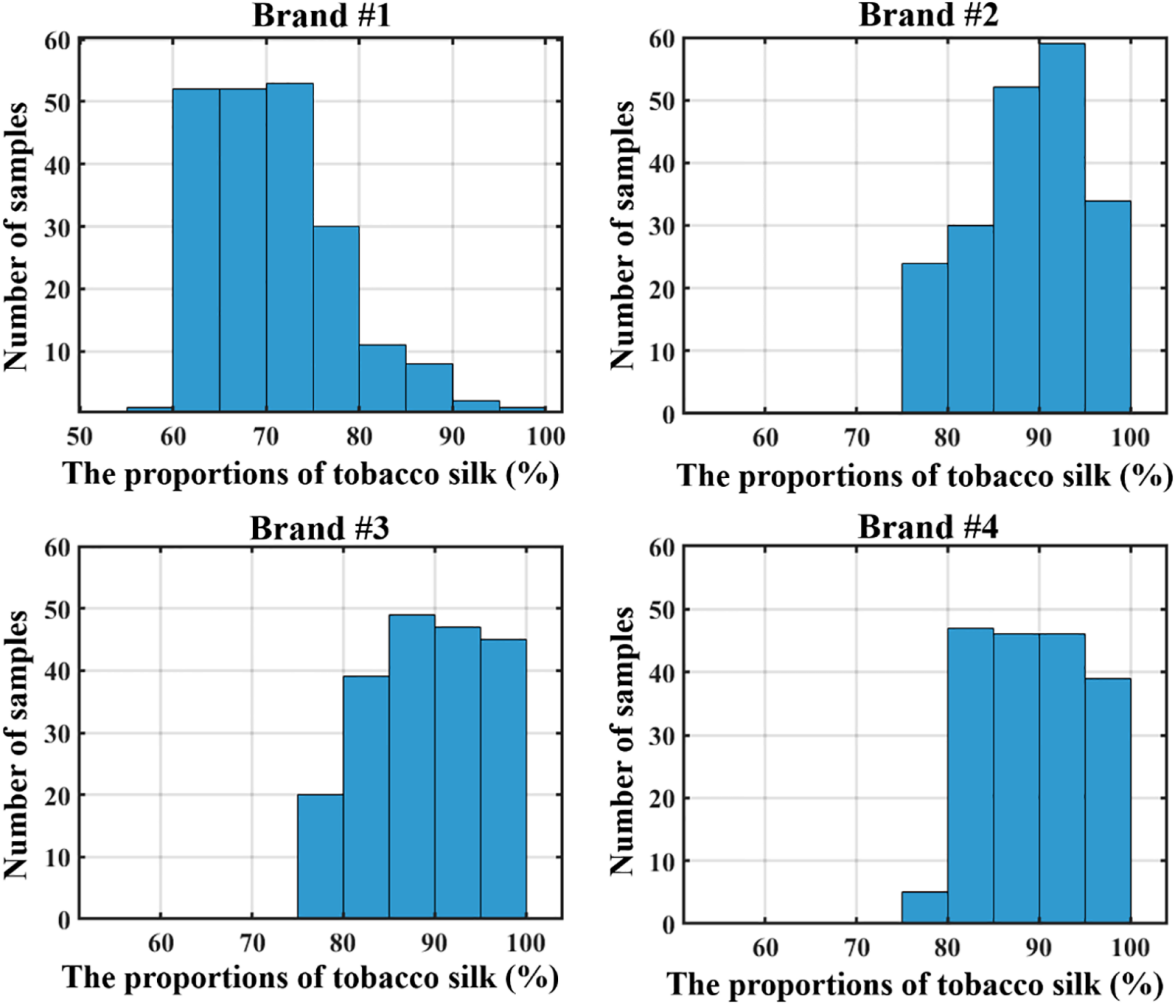
Distribution of tobacco silk blending proportions in formulated tobacco across different cigarette brands (Note: each column corresponds to a 5% interval of tobacco silk proportion).

**Table 2** summarizes the descriptive statistical data of tobacco silk blending proportions in formulated tobacco samples across different cigarette brands, including the mean, maximum, minimum, standard deviation, and coefficient of variation for each brand. **Figure 1** illustrates the distribution patterns of tobacco silk blending proportions for each brand. It can be observed that Brand #1 exhibits the widest distribution range, with values primarily concentrated between 60% and 80%. In contrast, the blending proportions for Brands #2, #3, and #4 are predominantly distributed within the 75%–100% range. Notably, Brand #2 and Brand #3 display highly similar distribution characteristics, with average blending proportions around 88.9% and coefficients of variation close to 7%.

**Table 2:** Statistical information on the blending proportions of tobacco silk in formulated tobacco across different cigarette brands.

Brand	Min(%)	Max(%)	Mean(%)	SD(%)	CV
#1	59.996	96.718	70.682	7.452	10.543%
#2	75.532	100.000	88.921	6.202	6.975%
#3	75.085	99.912	88.985	6.510	7.316%
#4	77.226	99.862	89.513	5.743	6.416%

### 3.2 Spectral diversity of formulated tobacco from different cigarette brands

**Figure 2:**
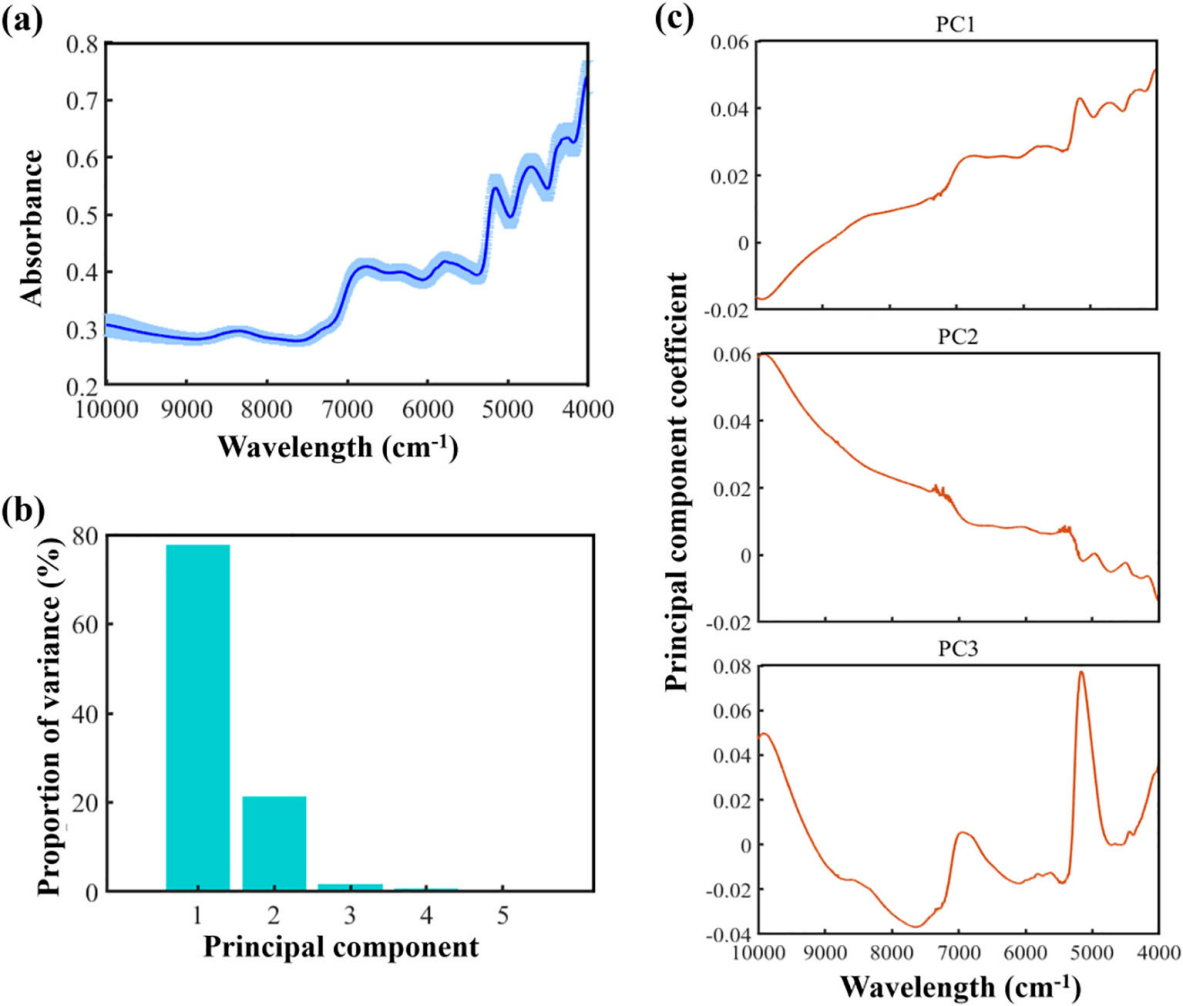
Spectral diversity of formulated cut tobacco: **(a)** Mean absorbance and standard deviation across all brands; **(b)** Variance explained by the first five PCs of spectra; **(c)** The principal component coefficient of the first three PCs.

An initial analysis was conducted on the near-infrared spectra of formulated tobacco samples across all cigarette brands. As illustrated in **Figure 2a**, the spectral profiles exhibited similar overall trends across brands, with consistent positions for spectral peaks (6780 cm^-1^, 5760 cm^-1^, 5130 cm^-1^, 4700 cm^-1^, 4280 cm^-1^) and troughs (6055 cm^-1^, 5360 cm^-1^, 4950 cm^-1^, 4500 cm^-1^, 4160 cm^-1^). Specifically, the spectral region around 5760 cm^-1^ is associated with the stretching vibrations of C–H bonds in aromatic compounds (Salzer, 2008), while the region near 5130 cm^-1^ corresponds to C=O stretching vibrations commonly found in esters and acids (Salzer, 2008). The region from 4000 to 4800 cm^-1^ is primarily related to absorption by CH, NH, and OH functional groups, and the range of 5725–6110 cm^-1^ is dominated by first overtone stretching vibrations of CH and SH groups—spectral features that are often linked to key components of formulated tobacco such as sugars and nicotine (Bi et al., 2019). Observable spectral differences across all samples are largely attributed to their underlying physical and chemical properties, which form the basis for detecting tobacco silk blending proportions using spectral data.

PCA was performed on the merged spectral dataset across all brands. The results revealed that the first three PCs accounted for 99.55% of the total variance, with PC1, PC2, and PC3 contributing 77.35%, 20.91%, and 1.29%, respectively (**Figure 2b**), indicating that these two components capture the majority of the spectral variability in formulated tobacco. As shown in **Figure 2c**, PC1 exhibited high positive coefficients in the 4000–7062 cm^-1^ range, suggesting a strong positive correlation and notable spectral variation in this region, which is consistent with the observed spectral trends in **Figure 2a**. This region is commonly associated with the absorption characteristics of organic substances such as moisture and proteins. PC2 demonstrated high positive coefficients in the 7380–10000 cm^-1^ range, indicating that this spectral region is closely associated with PC2 and also exhibits substantial spectral variation. PC3 exhibits a relatively high principal component loading around the 5000 cm^-1^ region. Collectively, the first three PC effectively captured the majority of the relevant information embedded in the spectral dataset.

**Figure 3:**
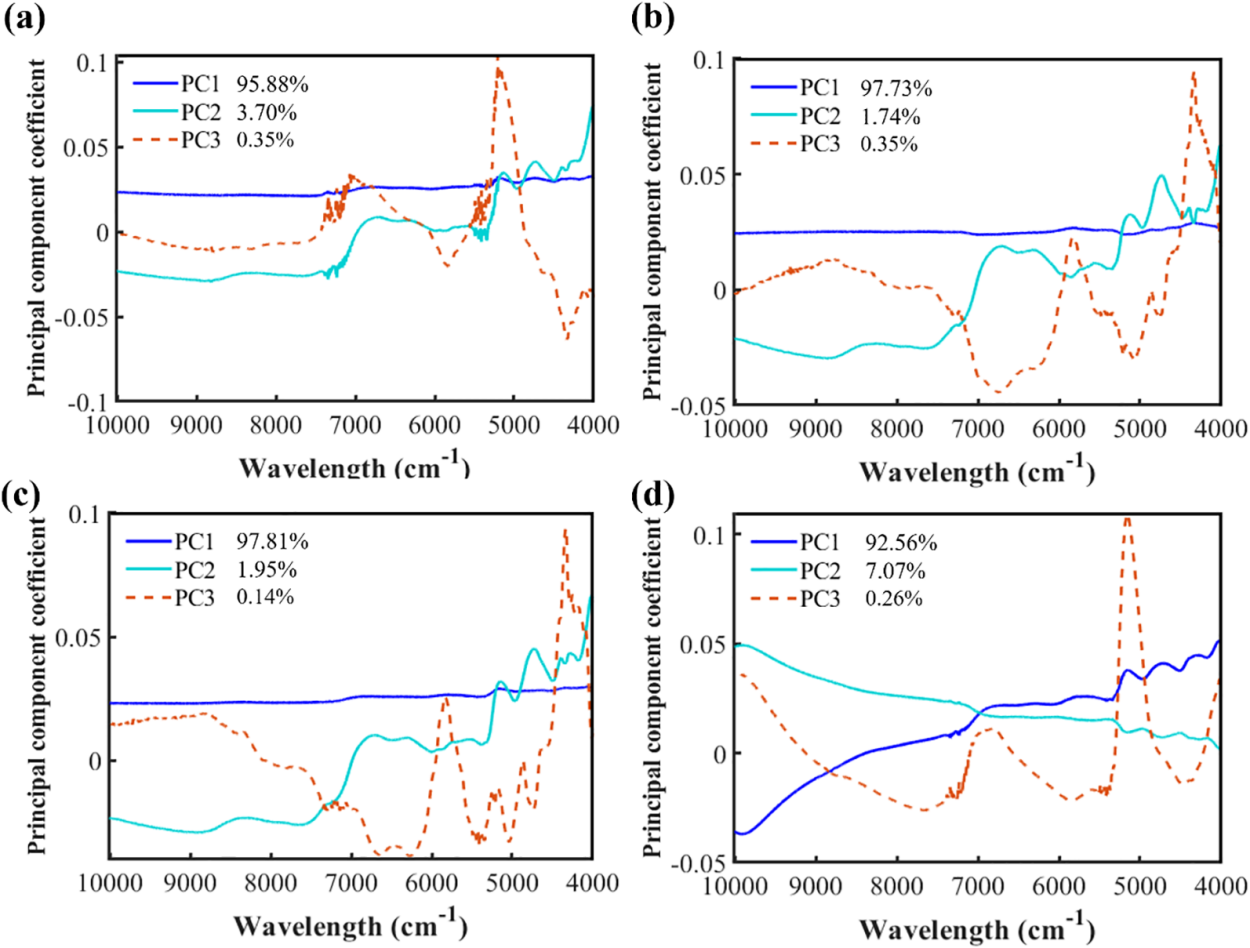
The first three PC with the highest variance contribution in the spectra of formulated tobacco from different cigarette brands: **(a)** Brand #1, **(b)** Brand #2, **(c)** Brand #3, **(d)** Brand #4.

**Figure 3** presents the PCA results of the spectra for formulated tobacco from different cigarette brands. Subfigures (a) to (d) correspond to the first three PC coefficients for Brands #1, #2, #3, and #4, respectively. For all brands, the cumulative variance explained by the first three PC exceeds 99%, indicating that these components effectively capture the majority of spectral variation across brands. The spectral feature distributions vary among brands. For Brands #1, #2, and #3, PC1 accounts for more than 95% of the total variance and exhibits similar trends, with PC1 coefficients remaining relatively stable at around 0.25 across the entire spectral range. In contrast, the PC1 of Brand #4 displays a steadily increasing trend over the same region. Regarding PC2, the overall patterns for Brands #1, #2, and #3 are relatively similar; Brand #1 shows a higher variance explained by PC2 (3.70%) compared to Brand #2 (1.74%) and Brand #3 (1.95%). Moreover, the PC2 coefficients of Brand #1 exhibit notable fluctuations around 7200 cm^-1^ and 5300 cm^-1^. In comparison, Brand #4 shows the highest variance contribution for PC2 at 7.07%, with PC2 coefficients gradually decreasing across the spectral range. For PC3, the differences among brands are most pronounced: Brands #2 and #3 share a similar trend, whereas Brands #1 and #4 display distinctly different patterns. These observations further highlight the spectral diversity among formulated tobacco samples from different cigarette brands.

### 3.3 Modeling tobacco silk proportions based on combined data from all brands

**Figure 4:**
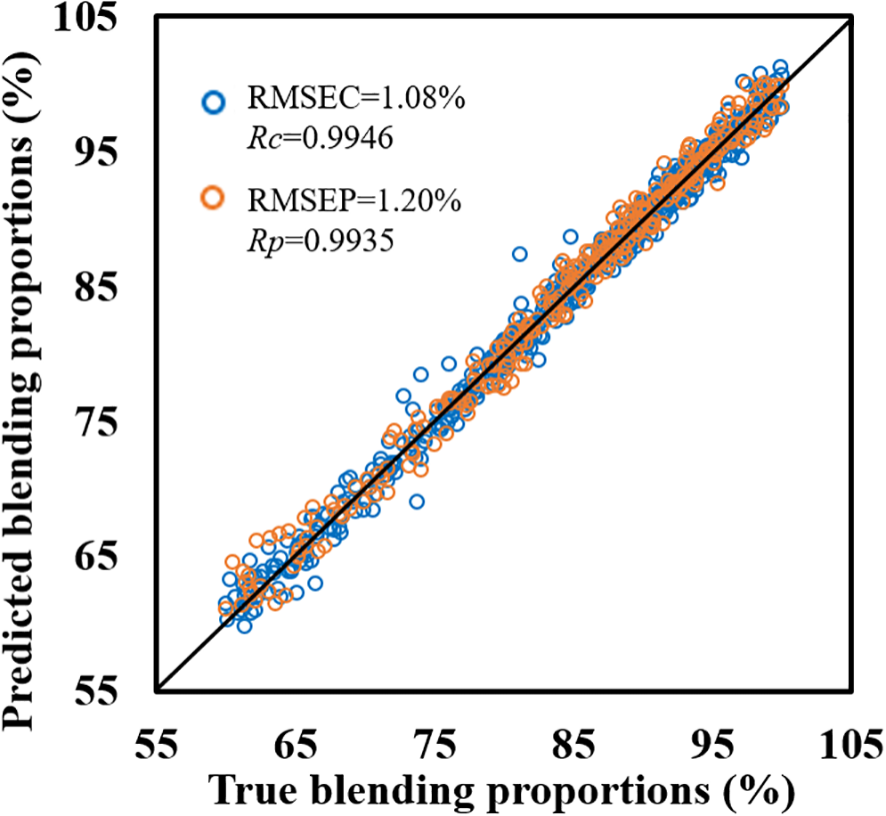
Results of the tobacco silk blending proportions detection model based on all cigarette brands.

To evaluate the feasibility of using NIRS for detecting the blending proportions of tobacco silk in formulated tobacco, a PLSR model was developed using spectral data and blending proportions from all four cigarette brands. A total of 792 samples were included for modeling and further analysis. Following the procedure described in section 2.4, the complete dataset was partitioned into calibration set and prediction set. PLSR model was constructed based on the calibration set, with the optimal number of latent variables determined via 10-fold cross-validation. The resulting model was then applied to the prediction set. As shown in **Figure 4**, both *R_c_* and *R_p_* reached 0.99, while RMSEC and RMSEP were as low as 1.08% and 1.20%, respectively. These results demonstrate the feasibility of establishing a reliable NIRS-based model for quantifying tobacco silk blending proportions in formulated tobacco. Despite the promising performance, it should be noted that samples in the calibration set and prediction set both originated from the dataset that included all four brands. In practical scenarios, however, the model is often expected to generalize to other brands not involved in the model development. As discussed in sections 3.1 and 3.2, there are notable differences in both tobacco silk blending proportions and spectral features among different brands. Therefore, it is necessary to further investigate the model’s adaptability and transferability to ensure its robustness and applicability across diverse cigarette brands.

### 3.4 Model transferability between different brands

#### 3.4.1 Performance of PLSR on transfer tasks

The performance of PLSR models on transfer tasks, in which a single brand was used as the calibration set (source domain) and a different brand was used as the prediction set (target domain), is summarized in **Table 3**. It can be observed that the model established based on the source domain brand exhibits performance differences when applied to the target domain brand, reflecting the impact of distributional discrepancies between datasets on the model’s generalization ability. Overall, all models achieved high prediction accuracy on the source domains, with RMSEC below 0.80%. However, when applied to the target domain, the model’s performance declined to varying degrees.

**Table 3:** Performance of PLSR models on transfer tasks across different brands.

Transfer tasks	Source domain		Target domain		
	*R_c_*	RMSEC (%)	*R_p_*	RMSEP (%)	MAE (%)
#1→#2	0.9953	0.72	0.8175	26.76	26.36
#1→#3	0.9960	0.67	0.9388	36.12	35.96
#1→#4	0.9953	0.72	0.1823	47.31	33.48
#2→#1	0.9997	0.15	0.4753	9.76	7.92
#2→#3	0.9997	0.14	0.9982	1.96	1.92
#2→#4	0.9996	0.17	0.7759	10.64	9.91
#3→#1	0.9998	0.14	0.5000	10.43	8.49
#3→#2	0.9996	0.19	0.9991	1.11	1.07
#3→#4	0.9995	0.20	0.3541	12.89	7.08
#4→#1	0.9929	0.68	0.5669	17.83	16.62
#4→#2	0.9977	0.39	0.9955	10.61	10.59
#4→#3	0.9901	0.78	0.9964	9.72	9.65

#1–#2 indicates that the model was developed using brand #1 and applied to estimate the tobacco proportions in brand #2. Similarly, #1–#3, #1–#4, and so on follow the same logic.

When Brand #1 was used as the source domain to build the model and transferred to Brand #2, Brand #3, and Brand #4, the model exhibited poor performance, with RMSEP values of 26.76%, 36.12%, and 47.31%, respectively. These results suggest substantial differences in feature distributions between Brand #1 and the other datasets, leading to decreased prediction performance. The RMSEP values on the target domain also reflect, to some extent, the degree of distributional discrepancy between brands. Notably, the highest RMSEP was observed for the transfer from Brand #1 to Brand #4, indicating the largest feature divergence between these two datasets. When Brand #2 was used as the source domain, the model achieved favorable prediction performance on Brand #3, with an *R_p_* of 0.9982 and a low RMSEP of only 1.96%, indicating that the feature distributions between Brand #2 and Brand #3 are relatively similar, thereby enabling the model to generalize well to the target domain. In contrast, transfers from Brand #2 to Brand #1 and Brand #4 resulted in higher RMSEP values of 9.76% and 10.64%, respectively, reflecting relatively poorer generalization performance. Similarly, when Brand #3 served as the source domain, the transfer to Brand #2 yielded good performance (RMSEP = 1.11%), consistent with the reverse direction (Brand #2 → Brand #3). However, transfers from Brand #3 to Brand #1 and #4 were less effective, with RMSEP values exceeding 10%. When Brand #4 was used as the source domain, the model demonstrated suboptimal transfer performance to all other datasets, with RMSEP values consistently greater than 9%. In particular, the transfer to Brand #1 yielded an RMSEP of 17.83%, suggesting a pronounced difference in feature distribution between Brand #4 and Brand #1.

In summary, in a few transfer tasks (e.g., Brand #2 → #3 and Brand #3 → #2), the model demonstrated satisfactory predictive performance on the target domain (RMSEP < 2%), suggesting high similarity in data distributions and effective direct model transfer. However, for the majority of transfer tasks, the direct application of models across brands resulted in considerable prediction bias, revealing the detrimental effects of feature distribution shifts on transfer learning performance. Therefore, it is essential to explore appropriate model transfer strategies to enable models trained on a single brand to be effectively applied to other brands.

#### 3.4.2 Performance of TCA-PLSR and Coral-PLSR on transfer tasks

To improve the cross-domain adaptability of the model, TCA and Coral were applied to transform the spectra before establishing the PLSR model, and the results were compared with the PLSR model without any transfer strategy. The results are shown in **Table 4**. Compared to the PLSR model (**Table 3**), the TCA-PLSR model demonstrates markedly improved performance in most of the transfer tasks, except for the Brand #2 → #3 and Brand #3 → #2 tasks, with both RMSEP and MAE showing varying degrees of reduction. In the transfer tasks of Brand #3 → #4 and Brand #4 → #3, compared to the PLSR model, the RMSEP of TCA-PLSR decreased by 87.98% and 89.09%, respectively, indicating that TCA effectively reduced the distribution deviation between Brand #3 and Brand #4 through subspace alignment, thereby enhancing the model’s cross-domain adaptability. Furthermore, in the mutual transfer tasks between Brand #1 and Brand #2, Brand #1 and Brand #3, and Brand #2 and Brand #4, TCA-PLSR outperforms both the PLSR and Coral-PLSR models in terms of RMSEP and MAE, further validating the robustness and transfer effectiveness of TCA-PLSR in most tasks.

**Table 4:** Performance of TCA-PLSR and Coral-PLSR models on transfer tasks across different brands.

Model	Transfer tasks	Source domain		Target domain		
		*R_c_*	RMSEC (%)	*R_p_*	RMSEP (%)	MAE (%)
TCA-PLSR	#1→#2	0.8884	3.41	0.6162	14.85	14.39
	#1→#3	0.9945	0.78	0.6810	18.22	17.43
	#1→#4	0.8912	3.37	0.4160	18.76	17.84
	#2→#1	0.9894	0.90	0.5171	6.46	6.98
	#2→#3	0.9994	0.22	0.9974	2.89	7.77
	#2→#4	0.9986	0.32	0.7272	6.58	7.16
	#3→#1	0.9917	0.83	0.4736	7.43	8.16
	#3→#2	0.9993	0.25	0.9980	0.64	6.97
	#3→#4	0.9991	0.27	0.9643	1.55	6.37
	#4→#1	0.9862	0.95	0.3548	10.95	10.38
	#4→#2	0.9910	0.77	0.9900	1.67	6.31
	#4→#3	0.9918	0.73	0.9918	1.06	7.70
Coral-PLSR	#1→#2	0.9953	0.72	0.8170	16.30	15.64
	#1→#3	0.9960	0.67	0.9400	25.06	24.84
	#1→#4	0.9953	0.72	0.2210	91.23	86.82
	#2→#1	0.9997	0.15	0.4988	16.42	14.92
	#2→#3	0.9996	0.17	0.9982	0.90	0.81
	#2→#4	0.9997	0.15	0.8601	15.40	15.12
	#3→#1	0.9995	0.20	0.5234	16.47	15.04
	#3→#2	0.9996	0.19	0.9991	0.49	0.43
	#3→#4	0.9999	0.07	0.7631	18.25	17.77
	#4→#1	0.9991	0.24	0.6015	27.20	26.49
	#4→#2	0.9991	0.24	0.9933	7.28	7.25
	#4→#3	0.9929	0.68	0.9969	6.02	5.84

#1–#2 indicates that the model was developed using brand #1 and applied to estimate the tobacco proportions in brand #2. Similarly, #1–#3, #1–#4, and so on follow the same logic.

In the transfer tasks of Brand #2 → #3 and Brand #3 → #2, Coral-PLSR demonstrated superior performance, compared to the PLSR model, RMSEP was reduced by 54.08% and 55.86%, respectively, accompanied by substantial decreases in MAE to 0.81% and 0.43%, which demonstrates the effectiveness of Coral-PLSR in certain model transfer tasks. It is noteworthy that in the transfer tasks of Brand #1 → #4, Coral-PLSR resulted in markedly poor performance, with RMSEP and MAE reaching as high as 91.23% and 86.82%, respectively, substantially higher than those of PLSR (47.31% and 33.48%). The application of Coral in this case adversely affected the model’s predictive accuracy on the target domain. Combined with the spectra analysis presented in Section 3.2, it is observed that the spectral features of Brand #2 and Brand #3 are similar, whereas the spectral features of Brand #1 and Brand #4 differ substantially. This suggests that Coral may not be suitable for model transfer tasks involving datasets with large distributional discrepancies.

The performance differences observed among the transfer learning methods can be attributed to their underlying mechanisms. TCA outperformed particularly in tasks where the feature distribution between the source and target domains differs markedly. This is probably because TCA facilitates better feature correspondence and knowledge transfer, thereby enhancing model adaptability across heterogeneous domains by aligning the marginal distributions in the projected space. In contrast, Coral-PLSR is more effective in scenarios where the source and target domains exhibit relatively similar distributions. Coral performs domain adaptation by aligning the covariance matrices of the source and target domains, under the assumption that their mean values are already comparable. This approach has a lower computational complexity compared to TCA and is less prone to overfitting when the domain gap is small. However, Coral is also more sensitive to sample perturbations and may fail to fully correct domain discrepancies when the underlying feature distributions are substantially different. Therefore, TCA is more suitable for transfer tasks with large distribution shifts, while Coral is preferable in cases with moderate or minimal domain differences.

In summary, TCA-PLSR performs more stably in most tasks and effectively reduces transfer errors, while Coral-PLSR demonstrates superior transfer effects in a few specific tasks. This result indicates that different transfer strategies have their applicability when addressing different source-target domain pairs, and the optimal strategy should be selected based on the data distribution of the specific transfer task to improve the model’s adaptability and accuracy.

#### 3.4.3 Performance of updated-PLSR, updated-TCA-PLSR, and updated-Coral-PLSR on transfer tasks

As discussed in section 3.4.2, TCA-PLSR and Coral-PLSR each exhibit superior performance in different transfer tasks, indicating that their effectiveness varies depending on the specific source–target domain pairs. To further assess the feasibility of improving transfer performance, this study explores the integration of model updating with TCA and Coral across model transfer scenarios.

**Figure 5:**
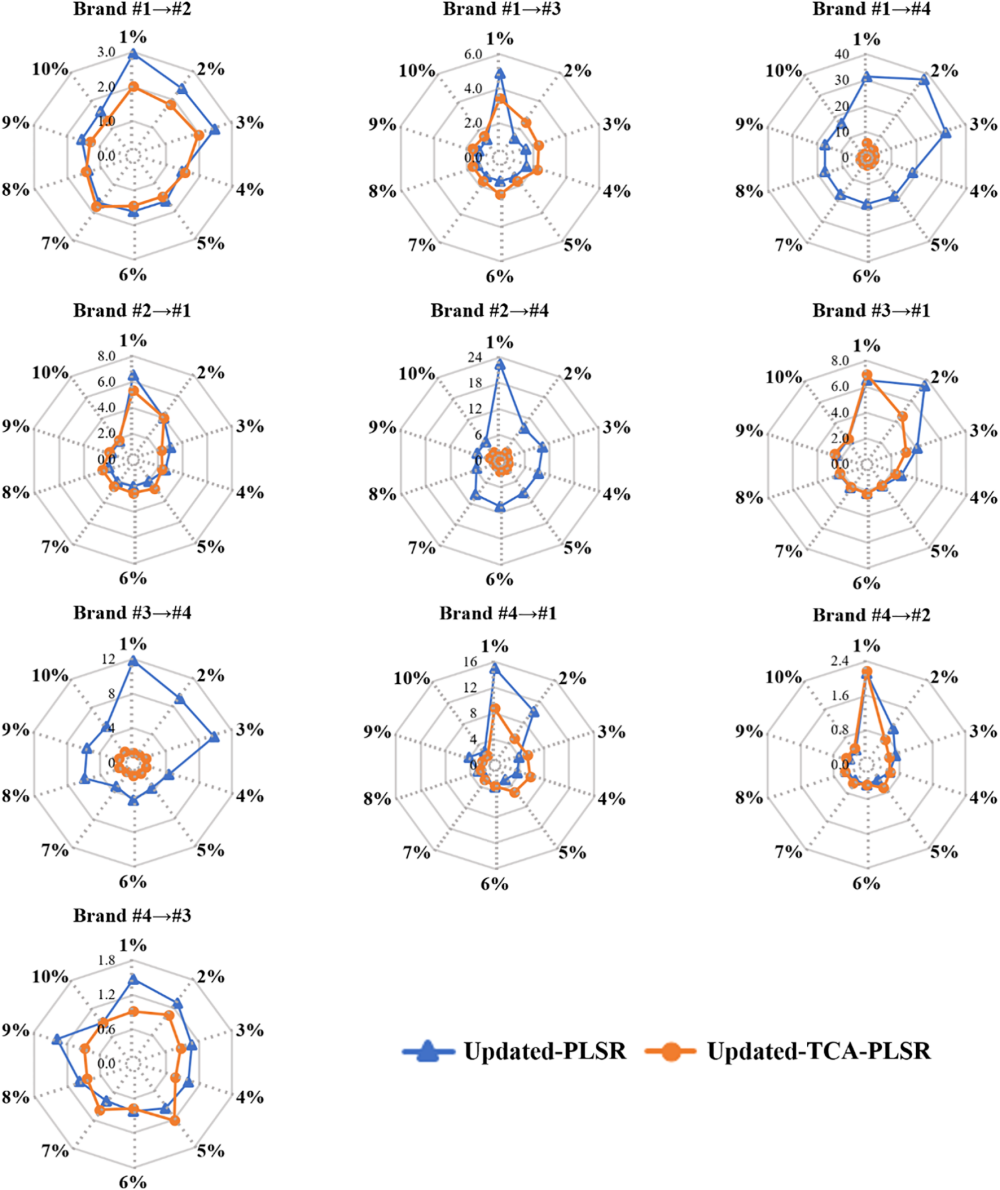
RMSEP of Updated-PLSR and Updated-TCA-PLSR when introducing 1%-10% samples of the target domain to the source domain (Note: the percentage represents the proportion of target domain samples added to the source domain relative to the total number of target domain samples).

**Figure 5** illustrates the RMSEP of the Updated-PLSR and Updated-TCA-PLSR when incorporating 1% to 10% of target domain samples into the source domain across 10 transfer tasks. Overall, the RMSEP of both Updated-PLSR and Updated-TCA-PLSR generally decreases with the increasing proportion of target samples introduced and tends to stabilize when the added proportion exceeds 6%, indicating that model updating effectively improves transfer prediction performance. However, in tasks characterized by substantial distributional divergence between source and target domains, the RMSEP of Updated-PLSR remains relatively high even after incorporating additional samples. For instance, in the Brand #1 → #4 task, the RMSEP of Updated-PLSR remains elevated at 16.62% after adding 10% of target samples, while the RMSEP of Updated-TCA-PLSR drops to 2.06%, demonstrating that the integration of TCA with model updating enhances the model’s ability to adapt to distributional shifts and improves predictive accuracy. Similar advantages of Updated-TCA-PLSR over Updated-PLSR are observed in the Brand #2 → #4 and Brand #3 → #4 tasks. Moreover, in transfer tasks such as Brand #1 → #2, Brand #1 → #3, Brand #2 → #1, Brand #3 → #1, Brand #4 → #1, Brand #4 → #2, and Brand #4 → #3, Updated-TCA-PLSR consistently achieves lower RMSEP than Updated-PLSR when only 1% of target domain samples are used for model updating. As the proportion of target samples increases, the RMSEP of Updated-TCA-PLSR gradually approaches that of Updated-PLSR, suggesting that Updated-PLSR requires a larger amount of target domain data to reach comparable transfer performance. These findings highlight the advantage of combining model updating with TCA, particularly when dealing with substantial domain discrepancies.

**Figure 6:**
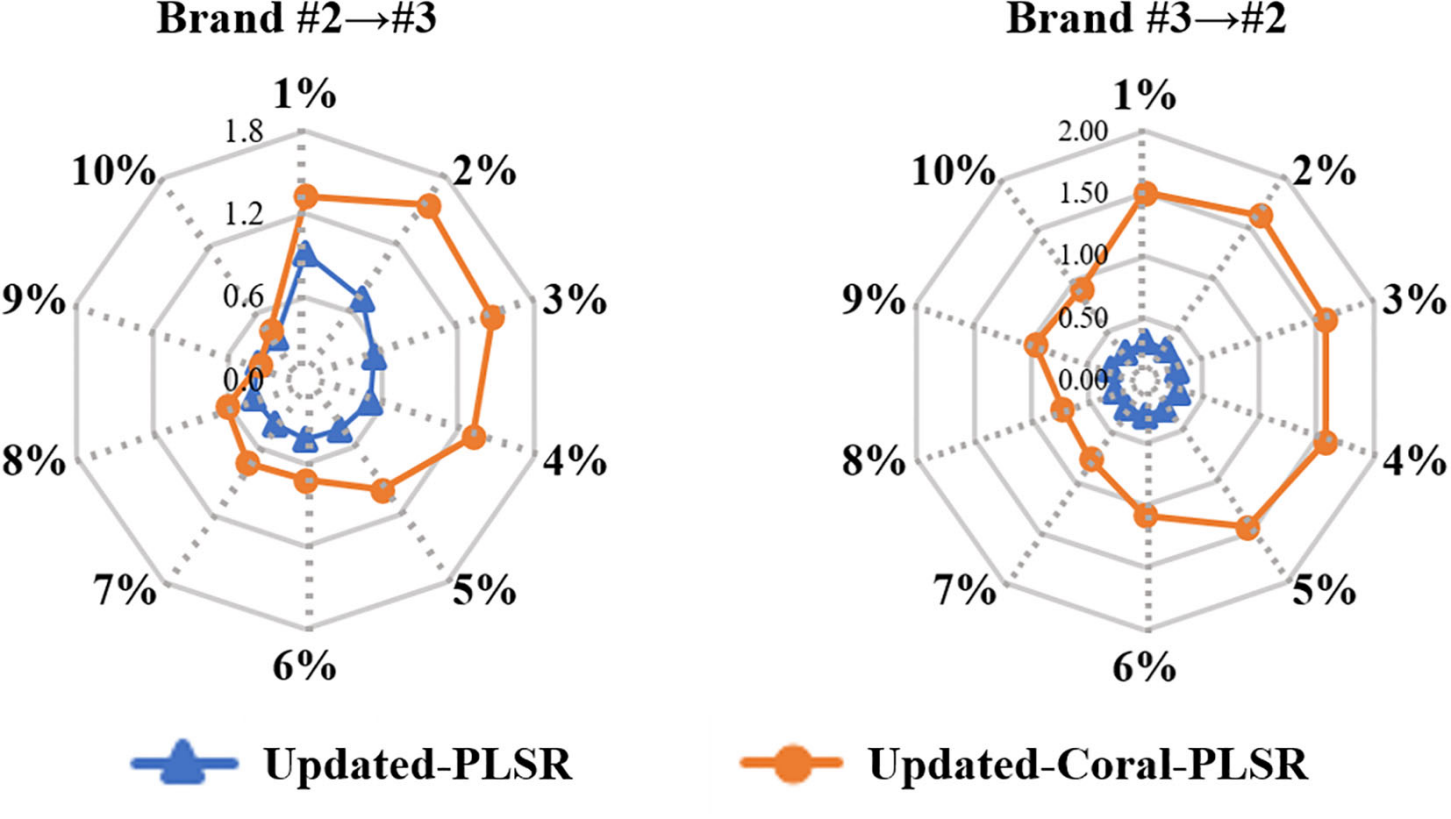
RMSEP of PLSR and Coral-PLSR when introducing 1%-10% samples of the target domain to the source domain (Note: the percentage represents the proportion of target domain samples added to the source domain relative to the total number of target domain samples).

**Figure 6** shows the RMSEP of Updated-PLSR and Updated-Coral-PLSR when 1% to 10% of target domain samples were incorporated into the source domain in the Brand #2 → #3 and Brand #3 → #2 transfer tasks. The two methods exhibit distinct RMSEP trends as the proportion of added samples increases. For Updated-PLSR, RMSEP decreases steadily with the inclusion of more target domain samples. In contrast, Updated-Coral-PLSR shows an initial increase in RMSEP at lower sample proportions (1%–4%), followed by a notable decrease once the proportion reaches 5% or higher. In particular, for the Brand #2 → #3 task, Updated-Coral-PLSR outperforms Updated-PLSR when 10% of the target domain samples are added. This suggests that Updated-Coral-PLSR may require a larger proportion of target domain data in the source domain to effectively perform feature alignment and achieve improved prediction performance. However, across most sample proportions, the RMSEP of Updated-Coral-PLSR remains higher than that of Updated-PLSR. As analyzed in Section 3.2, the spectral feature distributions of Brand #2 and Brand #3 are relatively similar, indicating that when the distributional divergence between domains is small, Updated-PLSR can more effectively learn the target domain characteristics. In such cases, applying Coral may introduce unnecessary transformations, thereby degrading model performance.

## 4 Conclusion

This study proposes a method for detecting the blending proportions of tobacco silk in tobacco formulations based on NIRS and explores the feasibility of transfer learning strategies to improve the model’s generalization capability. The results indicated that establishing a detection model for the blending proportions of tobacco silk in tobacco formulations using NIRS is feasible. However, when the model is applied to detect different cigarette brands, it performs poorly, suggesting that data distribution differences have a significant impact on the model’s adaptability. Therefore, this study investigates the feasibility of using TCA and Coral strategies to enhance model transfer performance, with TCA-PLSR demonstrating good performance in most model transfer tasks and improving the model’s cross-domain applicability. Furthermore, by introducing a small number of target domain samples to update the model, the cross-domain detection accuracy of the Updated-TCA-PLSR model is further improved. Overall, the method proposed in this study provides a viable technical solution for efficient and accurate quality evaluation across different cigarette brands, with significant implications for the intelligent detection of the tobacco industry. Future research could cover more cigarette brands and further integrate deep learning methods to enhance the model’s adaptability, enabling broader industrial applications.

## Data Availability

The raw data supporting the conclusions of this article will be made available by the authors, without undue reservation.
